# Predictors of Burnout Among Physicians: Evidence From a National Study in Portugal

**DOI:** 10.3389/fpsyg.2021.699974

**Published:** 2021-10-01

**Authors:** Alexandra Marques-Pinto, Sérgio Moreira, Rui Costa-Lopes, Nídia Zózimo, Jorge Vala

**Affiliations:** ^1^Centro de Investigação em Ciência Psicológica (CICPSI), Faculdade de Psicologia, Universidade de Lisboa, Lisbon, Portugal; ^2^Instituto de Ciências Sociais, Universidade de Lisboa, Lisbon, Portugal; ^3^Centro Hospitalar Lisboa Norte, Lisbon, Portugal

**Keywords:** burnout, physicians, organizational demands and resources, context variables, national study, predictors

## Abstract

The aims of this research on burnout among physicians were threefold, (1) to characterize the burnout symptoms’ prevalence among Portuguese physicians, (2) to test the hypothesis that organizational demands and resources add, on top of other factors, to the explanatory level of burnout; and (3) to explore the predictors of organizational demands and resources. Data collection was conducted online at the national level in Portugal, with 9,176 complete replies and a response rate of 21%. Predictors stemming from theoretical models of an intra-individual, occupational, organizational, and socio-psychological nature were measured using an online/paper survey. Results were analyzed through a significantly modified version of the Maslach Burnout Inventory (MBI) after transformations to address the fit of this measure in this sample. Results show that 66% of physicians have high levels of emotional exhaustion, 33% high levels of depersonalization, and 39% high levels of decrease of personal accomplishment. Moreover, a first set of hierarchical multiple regression models with burnout symptoms reveals that organizational resources, demands of the relationship with the patients and of work schedule are consistently important predictors of emotional exhaustion and depersonalization on top of other theoretically relevant predictors. A second set of regression models with the organizational-level variables shows that, aside from organizational variables, other context variables, like procedural justice and teamwork, have the most substantial predictive value. These results highlight the importance of recognizing physicians’ burnout as a phenomenon that is predicted by a wide variety of factors, but also the importance of attending to the particular role of circumstancial factors that may be addressed in future interventions.

## Introduction

Physicians’ burnout is recognized as a public health problem (e.g., West et al., 2018) due to its purportedly high prevalence (Schwenk and Gold, 2018) and implications for individual physicians and their families’ well-being (Shanafelt et al., 2015), the quality of patient care and the costs it brings to health organizations and systems (Panagioti et al., 2017). Burnout has long been defined as a syndrome of emotional exhaustion, depersonalization, and low sense of personal accomplishment at work that is driven by prolonged professional stress (Maslach, 1976) and tends to become a chronic condition (Schaufeli and Enzmann, 1998). With this regard, Guthier et al. (2020) recent meta-analysis has unraveled that job stressors and burnout mutually affect each other, and called for new job stress interventions that also promote ways of coping with the burnout symptoms, with a view to prevent this possible vicious circle.

Although the Maslach (1976) three-dimensional definition of the burnout syndrome is somewhat consensual within the literature and the Maslach Burnout Inventory (MBI; Maslach et al., 1996) is regarded as the gold standard measure of burnout, there is still some controversy among burnout’s authors regarding the definition and assessment of the burnout symptoms. Indeed, there is a persistent debate about the “centrality” of the different dimensions in the burnout definition, leading some authors to assess burnout solely through measures of emotional exhaustion (e.g., the Copenhagen Burnout Inventory, Kristensen et al., 2005). Nevertheless, according to Schaufeli et al. (2009, pp. 211), “there is no scientific reason to use the term, burnout, when referring to exhaustion only” and this reductionist view of burnout as exhaustion-only may contribute to measurement artifacts such as cross-sectional associations between exhaustion (i.e., work-related distress) and work-related stress measures (Schaufeli and Enzmann, 1998), specially when the items of both types of measures show content overlap (Bianchi et al., 2021).

In the health care professions, namely in the medical profession (West et al., 2009; Shanafelt et al., 2012), burnout is often defined and measured by assessing the frequency of symptoms in two domains, emotional exhaustion, and depersonalization (Hewitt et al., 2020), which are considered as the “core of burnout” (Green et al., 1991). In fact, the validity of the personal accomplishment/efficacy scale has been questioned (e.g., Bresó et al., 2007), even among the authors who use the MBI (Maslach et al., 1996). Due to its positively framed items which are later reversed, and to some theoretical frameworks of burnout that position this dimension as a personal predisposition which may impact the development of the other dimensions of burnout (i.e., emotional exhaustion and depersonalization/cynicism) (Bresó et al., 2007), some authors do not consider this dimension at all in burnout studies (e.g., Ancona and Mendelson, 2014). Currently there seems to be a broad agreement in the literature that exhaustion and depersonalization represent the core symptoms of burnout, the former having reciprocal relations with job stressors and the latter having no direct links with job stressors, according to Guthier et al. (2020) meta-analysis. Moreover, despite the recognized utility of the MBI (Maslach et al., 1996) and its extended use in studies of physicians, short versions such as single item measures of emotional exhaustion and depersonalization have also proven to be useful in assessing burnout in medical professionals when the full MBI cannot be applied (e.g., West et al., 2009).

Not surprisingly, the high heterogeneity found in burnout research precludes clear conclusions about the prevalence of physicians’ overall burnout or burnout symptoms (e.g., Rotenstein et al., 2018; Hewitt et al., 2020). Some national studies of burnout among large samples of United States and European physicians have been published in the last decade suggesting that this may be a phenomenon of epidemic proportions that affects half to two-thirds of practicing physicians (Schwenk and Gold, 2018). For example the United States national studies, covering all specialties, by Shanafelt et al. (2012, 2015) revealed that 46 and 54% of the physicians presented high scores in emotional exhaustion and/or depersonalization. Similar data from other countries are not widely available (West et al., 2018) but the literature reflecting large-scale studies within specific specialties, such as, in Europe, the European General Practice Research Network (EGPRN) study (Soler et al., 2008), suggests that this phenomenon is present worldwide. Nevertheless, Rotenstein et al. (2018) systematic review showed 142 different definitions used for meeting burnout symptoms or overall burnout criteria and reported a large variability in prevalence estimates of physicians’ burnout, with emotional exhaustion ranging from 0 to 86.2%, depersonalization from 0 to 89.9%, low personal accomplishment from 0 to 87.1%, and overall burnout from 0 to 80.5% (Rotenstein et al., 2018). Also, most of these percentages were statistically based on frequency distributions and do not refer to consensual clinical external criteria (Maslach et al., 1996; Schaufeli et al., 2009; Bianchi, 2017), thus burnout’ prevalence cannot be clinically established.

Studies on Portuguese physicians’ burnout present similar problems of heterogeneity namely in the target populations and in the definitions of burnout used, since most of them have focused on different specialties (e.g., family doctors; Marcelino et al., 2012) and presented the MBI results as a continuous measure of burnout symptoms or of overall burnout, or as a dichotomous measure of burnout, thus hindering clear conclusions about the prevalence of burnout in this professional group at a national level in Portugal. Hence, as a first goal this study set out to characterize the three burnout symptoms prevalence among Portuguese physicians, at a national level and encompassing all specialties.

As regards the impacts of burnout, the literature in the field suggests that physicians who complain of burnout symptoms are more likely to report substance abuse, depression and suicide, broken familiar relationships, professional dissatisfaction, turnover and early retirement, among other problems (e.g., Dyrbye et al., 2017). Importantly, burnout in physicians has also been associated with higher frequency of medical errors (West et al., 2006), reduced quality of patient care and satisfaction (e.g., Panagioti et al., 2018) and increased health care costs (e.g., Dyrbye et al., 2017). Therefore, physicians’ burnout has caught the interest of researchers, practitioners, and health policy leaders in establishing its main determinants, with a view to further understand the phenomenon and to inform interventions aiming to reduce the risk of burnout in physicians (e.g., West et al., 2016; Panagioti et al., 2017). Nevertheless, these issues are far from being uncontroversial (Schwenk and Gold, 2018).

For the past 45 years, two contrasting perspectives have been used to explain the development of burnout in diverse professional groups, a medical perspective that envisages burnout as a psychiatric condition and a psychosocial perspective that tends to define burnout as a form of chronic distress resulting from highly stressful work environments (Schaufeli et al., 2009). Although the former may contribute to an official medical diagnosis of burnout “that opens the gates of the welfare state with its compensation claims and treatment programs” (Schaufeli et al., 2009, pp. 214), the latter defines burnout as a socio-professional problem that carries a minimum stigma in terms of a psychiatric diagnosis and calls for socio-professional responses to this problem.

According to this psychosocial perspective, adopted in the present study, physicians’ burnout is viewed not only as an individual problem but rather as a problem of the health care organizations as a whole, whose leading drivers are rooted in the working environment and organizational culture (Panagioti et al., 2017). Within this frame of reference, the Job Demands-Resources (JD-R) model (e.g., Demerouti et al., 2001; Schaufeli and Bakker, 2004) is currently recognized as one of the leading burnout models. The JDR offers a heuristic about how job and personal characteristics influence professional burnout, and with special relevance its core dimensions, with long-term perceived excessive job demands leading to exhaustion and perceived lack of job and personal resources contributing mainly to an increase in depersonalization (Schaufeli and Taris, 2014). The JDR has stimulated a large body of research, namely in physicians (e.g., Hakanen et al., 2008; Houkes et al., 2008), with both cross-sectional and longitudinal evidence giving considerable support to the model predicted effects, in particular to the role of job demands in the explanation of burnout (Schaufeli and Taris, 2014).

As regards research findings on physicians’ burnout predictors, significant relationships have been found between physicians’ burnout and demographic characteristics (e.g., sex, age, relationship status, age of children, and partner occupation) (e.g., Houkes et al., 2011) as well as other intra-individual variables such as coping skills and personality traits (e.g., neuroticism; Rotenstein et al., 2020; optimism; Fowler et al., 2020). However, within the predominant psychosocial perspective of burnout, situational work-related factors assume particular importance in the explanation of physicians’ burnout prevalence (West et al., 2018). Variables related to the medical specialties, the type of practice (e.g., public/private, academic) or the payment models (e.g., performance-based/salaried) are examples of occupational factors that significantly correlate with physicians’ burnout. Notably, perceived excessive job demands like high workloads or clerical burdens, and low job resources such as low support from colleagues and leaders, low autonomy/control and low meaning at work are strong significant organizational factors associated with burnout amongst physicians (Lee et al., 2013; Dyrbye et al., 2017; West et al., 2018). Additionally, socio-psychological variables have also been found to predict burnout and wellbeing in organizational environments (Tyler, 1989; Steffens et al., 2017); in particular, professional identity (Jager et al., 2017), organizational justice (Jin et al., 2015), and perceived inequity (Smets et al., 2004) have significant relationships with physicians’ burnout.

Despite the important contributions from empirical research regarding physicians’ burnout main drivers, many gaps remain in this field of study (see Dyrbye et al., 2017 for a thorough discussion of research needs). Findings on the correlates of burnout as well as evidence from meta-analyses and longitudinal studies suggest that perceived organizational demands and resources are the most important predictors of professional burnout (e.g., Schaufeli and Buunk, 2003), namely that of physicians (e.g., Linzer et al., 2001). Nevertheless, as pointed out by West et al. (2018), studies have failed to take into consideration the full range of potential contributing factors and to determine their unique value in the explanation of physicians’ burnout. For instance, several important studies (e.g., Swider and Zimmerman, 2010; Bianchi et al., 2021) have shown the relevant role of stable personality traits such as neuroticism and other dimensions of the Five Factor Model (Goldberg, 1990) in the explanation of burnout, but there is still an “unwise” tendency to de-emphasize individuals’ personality factors in burnout research, with implications for the conceptualization and effective prevention of burnout (Bianchi et al., 2021). Supporting this claim, a study by Bianchi et al. (2021) exploring the associations between teachers’ burnout and an array of work-situated, work-unrelated, dispositional and intersecting variables, revealed that neuroticism, job strain, skill development, security in daily life, and work-non-work conflict were consistently linked to burnout. Moreover, results of relative weight analysis showed that neuroticism was the best predictor of teachers’ burnout.

Thus, and in a similar vein of Bianchi et al. (2021) study, further research is required to ascertain the unique predictive value of perceived organizational demands and resources in the explanation of burnout dimensions within a model that takes into account other relevant identified predictors of burnout, including intra-individual, personality factors. Hence, a second main goal of this study was to determine the predictive value of a myriad of occupational, socio-psychological, intra-individual and organizational factors in the explanation of physicians’ burnout. Within these four levels of analysis, the selection of factors was based on empirically supported and/or theoretical associations with physicians’ burnout, namely by focusing on other factors that have previously emerged as relevant in other indirectly-related domains. More specifically this study set out to test the hypothesis that perceived organizational demands and resources are strong significant predictors of physicians’ burnout, on top of the predictive value of the other factors. This idea has been suggested in the literature (e.g., Houkes et al., 2008) but has not yet been comprehensively tested (e.g., Dyrbye et al., 2017; West et al., 2018). Also, and according to the psychosocial perspective adopted in the present study, the organizational demands and resources are considered of particular importance since they provide context for practical interventions with broad implications across physicians in the workplace.

Following what we said above about the broad tendency found in the burnout research (see Guthier et al., 2020), combined with parsimony reasons, only the core dimensions of burnout, exhaustion and depersonalization, were used in these analyses. Additionally, this choice is in line with the JDR heuristic about the role played by organizational demands and resources in the explanation of both dimensions (Schaufeli and Taris, 2014).

Mirroring theory and research findings on physicians’ burnout drivers, some systematic reviews and meta-analysis studies on interventions aiming to reduce the risk of burnout in physicians (e.g., West et al., 2016; Panagioti et al., 2017) also emphasize the role of job demands and resources in explaining this syndrome. Nevertheless, additional studies are recommended with a view to disentangle what works best for whom, in which circumstances, that is to say, to unravel the factors at the participant, at the context, and at the intervention development and implementation levels that may account for differences in the effectiveness of interventions (West et al., 2016; Panagioti et al., 2017). This challenge is in line with a view of organizational interventions as active programs that dynamically interact with contextual factors and emerging processes, requiring, according to a realistic evaluation approach (Pawson, 2006), an integrated context-process-outcome evaluation framework (see Nielsen and Maglia, 2017 for a critical essay on this subject). Such an evaluation, namely of mechanisms/processes, is beyond the scope of this study but it is plausible, in line with Nielsen and Maglia (2017), that a way to increase intervention potential in the prevention of physicians’ burnout is to explore context factors.

Hence, this study goes upstream and sets as a third goal to explore the predictive value of occupational, socio-psychological, intra-individual, and organizational context variables in the explanation of the hypothesized best drivers of burnout among physicians, that is to say perceived organizational demands and resources. This enhanced theoretical understanding of the context variables that influence perceived job demands and resources is a specific contribution to the literature emerging from this study and it may contribute to the design and implementation of optimized organizational interventions and, therefore, to increase their effectiveness.

## Materials and Methods

### Sample

A total of 43,983 fully licensed physicians registered in the Portuguese Medical Association (OMP in Portuguese) and with eligible emails were individually invited *via* email to participate in the National Study on Physicians’ Burnout (NSPB). The 9,176 physicians that replied to the invitation composed the sample in this study, representing a response rate of 21%. To maximize the participation response rate of physicians, we developed a detailed communication plan including preparatory meetings with teams of physicians in the three Regional Sections of the Medical Association, promotion of the study in conferences, email remainders, and multiple ways to access and respond to the survey online. Comparisons of the distribution of gender, age, and regional affiliation to orthogonal matching pursuit (OMP) between the population, the study sample and an overlaid random sample showed very small differences between the study sample and the population (see Supplementary Table 1).

### Procedure

#### Survey

The survey was disseminated through Qualtrics using a private individual link to assure minimization of multiple responses by the same participant and by participants outside the study’ universe. More specifically, a private link was generated and sent individually by email to each of the 43,983 registered physicians with eligible emails in the OMP.

A total of 12,580 physicians initiated the survey and completed it up to the burnout symptoms section (representing a response rate to the burnout measure of 29%) of which 9,176 answered all questions and submitted the survey, resulting in a completion rate of 73%. An analysis of completion times (excluding extreme cases) reveled that on average participants took 40 min to complete the survey (SD = 20) and that half of the participants responded to the survey in less than 35 min.

#### Measures

As for the instruments selected to measure the study variables, the relevant literature was reviewed, namely reporting similar large-scale studies (e.g., Soler et al., 2008; Shanafelt et al., 2012, 2015), and validated scales were chosen whenever available and appropriate. For some of the scales a selection of the original list of items was made with a view to balance the length and the thematic broadness of the survey.

Statistical psychometric quality of validated scales was accessed using Confirmatory Factor Analysis for validated scales with two sets of criteria (Brown, 2006): measures of the overall Goodness of Fit (GFI) – Root Mean Squared Error of Approximation (RMSEA) <0.07 and Comparative Fit Index (CFI) and Tucker-Lewis Index (TLI) >0.90; and measures of localized areas of strain – standardized residuals <2.60 and general modification indexes <5. Statistical psychometric quality of customed or adapted scales was accessed using Exploratory Factorial Analysis and the following criteria for quality (Brown, 2006; Tabachnick and Fidell, 2007): factors’ eigenvalues >0.7, factorial weights >0.30, and communalities >0.09.

Statistical psychometric quality was assessed using RStudio version 1.3 with the package lavaan (Rosseel, 2012).

##### Occupational variables

The difference between effective and contracted working hours, compensatory rest after night shift in emergency, number of years as specialist, professional stability, work context, professional income, primary workplace, and number of workplaces (e.g., Soler et al., 2008) were measured.

##### Socio-psychological variables

Two dimensions already considered as important in the study of burnout and organizational wellbeing were considered: professional identity (Steffens et al., 2017), measured using item 5 (centrality), 4 (ingroup affection), and 2 (ingroup bonding) of Cameron (2004) identity scale (explained variance of 62%, factorial weights between 0.61 and 0.94, α = 0.80); and perceptions of justice (Tyler, 1989), specifically, procedural justice measured using a single item adapted from the European Social Survey (ESS Round 7: European Social Survey, 2015) and Professional deprivation measured using two items adapted from Lima and Vala (2004) Relative Deprivation Scale (bivariate correlation >0.30).

##### Organizational variables

A customized scale was developed for the NSPB study which covered a set of organizational resources and demands drawn from the general literature on these determinants of burnout (e.g., Schaufeli and Taris, 2014) and from the literature specifically focusing on physicians (e.g., Houkes et al., 2008; Lee et al., 2013). The details of the scale validation are described elsewhere (name deleted to maintain the integrity of the review process) and in the present study the factorial structure proposed by the authors was used, with a total of nine factors: organizational resources; demands of the relationship with suffering patients; mental demands; demands of the relationships (with colleagues or staff members) in the workplace; work schedule demands; physical demands; demands due to the lack of resources; demands of the relationship with patients (e.g., disrespectful) in general; and demands due to the lack of autonomy (explained variances ranged from 12 to 2%, factorial weights between 0.37 and 0.88, alphas between 0.90 and 0.68, with two factors composed by two items with bivariate correlations >0.30).

##### Intra-individual variables

Optimism, locus of control, self-efficacy, emotional regulation, problem-focused coping, and self-care were the intra-individual variables examined (e.g., Lee et al., 2013; Hojat et al., 2015; West et al., 2016):

• Optimism was measured using items 4, 9, and 10 of the Life Orientation Test-Revised (LOT-R; Scheier and Carver, 1985; Scheier et al., 1994; EFA with a single factor with 50% of explained variance, factorial weights between 0.37 and 0.87, and α = 0.69).
• Locus of control was measured using, items 13a, 13b, 28a, and 28b of the reduced version of the Rotter’s Locus of Control Scale (Rotter, 1966; EFA with a single factor with 35% explained variance, factorial weights between 0.50 and 0.71, and α = 0.66).
• Self-efficacy was measured using items 4, 5, and 10 of the Portuguese version of the Generalized Self-Efficacy Scale (Schwarzer and Jerusalem, 1995; Nunes et al., 1999; EFA with a single factor with 72% of explained variance, factorial weights between 0.78 and 0.93, and α = 0.88).
• Emotional regulation was measured using items 5 and 8 of the cognitive reappraisal and 4 and 9 of the emotional suppression subscales from the Portuguese version of the Emotion Regulation Questionnaire (ERQ; Gross and John, 2003; Vaz, 2009; bivariate correlations indicated that only the cognitive reappraisal items had *r* > 0.30 and consequently the emotional suppression items were dropped off the study).
• Problem-focused coping was measured using subscales of active coping and planification of the Portuguese version of Brief COPE (Carver, 1997; Pais Ribeiro and Rodrigues, 2004; EFA with a single factor with 73% of explained variance, factorial weights between 0.68 and 0.95, and α = 0.91).
• Finally, self-care was measured using level of satisfaction with physical activity, leisure time, sleep quality, and eating behaviors (Chambers et al., 2006; Renpenning and Taylor, 2011; EFA with a single factor with 52% of explained variance, factorial weights between 0.66 and 0.62, and α = 0.81).

##### Burnout symptoms

The MBI-HSS – Maslach Burnout Inventory Human Services Survey adapted for medical personel [Maslach et al., 1996, Portuguese version by Marques Pinto (2002)] was used to measure the three burnout dimensions: emotional exhaustion, depersonalization, and reduced personal accomplishment. Since the MBI is an extensively used scale, both internationally and in the Portuguese context, and its factorial structure has been consistently replicated, a Confirmatory Factorial Analysis (CFA) was used to test the MBI psychometric properties in the study sample. Items 9, 6, 10, 11, 12, 13, 14, 16, 19, and 21 were removed based on the modification indexes (all >5). The results for this modified version of MBI showed good adjustment for the tri-factorial version (factors for emotional exhaustion, depersonalization, and professional realization) with CFI = 0.95, TLI = 0.94, GFI = 0.96, and RMSEA = 0.07 and with alphas between 0.69 and 0.85. The results showed that emotional exhaustion has a correlation of 0.36 with depersonalization and 0.21 with reduced personal accomplishment, and that depersonalization has a correlation of 0.34 with reduced personal accomplishment.

##### Demographic variables

Gender, relationship status and age of the youngest child were considered as control variables, given their significant relationships with physicians’ burnout (e.g., Houkes et al., 2011).

#### Data Analysis

Hierarchical multiple linear regressions using a stepwise entry method were used to test the contribution of the different sets of variables to the explanation of the variation in the organizational resources and demands. Before the computation of the regression models, the conditions known to influence the computation of estimates were minimized. More specifically, the variables included in the study were scoped for the presence of response errors, outliers, frequency distributions, and multicollinearity (when applicable) and the linear models were tested for the adjustment of the distribution of the error to a normal distribution. Additionally, since the sample size may have a considerable influence on the computation of the test statistic values, a strict set of criteria were defined to interpret the results. This means that in the multiple regression analysis only standardized betas ≥0.10 and with *p*-values ≤0.01 were considered statistically significant (Cohen et al., 2003; Hemphill, 2003).

## Results

### Burnout Symptoms Prevalence Among Portuguese Physicians

The analysis of the mean values of the three burnout dimensions revealed values of 3.88 for Emotional exhaustion, of 1.40 for Depersonalization, and of 2.07 for reduced Professional accomplishment. Based on the MBI response scale, which ranged from 0 (Never) to 6 (Every Day), the Emotional exhaustion was the only burnout dimension above the midpoint of the scale. However, the analysis of the percentage of physicians scoring at a low, medium or high level in each of these indicators, considering the statistical cut-offs commonly used (Maslach et al., 1996) namely in the studies with physicians (e.g., Soler et al., 2008; Shanafelt et al., 2012, 2015) revealed that 66% of the study participants reported a high level of Emotional exhaustion, approximately 39% a high level of Depersonalization and about 30% a high decrease in their Personal accomplishment.

### Predictors of Burnout Symptoms

Two stepwise hierarchical regressions were computed using the core burnout dimensions, emotional exhaustion, and depersonalization, as dependent variables, demographic factors as control variables, and occupational, socio-psychological, intra-individual, and organizational variables as predictors, with each one of these five sets of variables added successively in the two regression models (Table 1; also see Supplementary Tables 3, 4). In order to test our main hypothesis regarding the explanatory power of job demands and resources on top of the other categories of variables, these variables were only introduced in the final step of each regression model.

**TABLE 1:** Effect sizes for the hierarchical multiple regressions on emotional exhaustion and depersonalization.

	Model 1^(a)^	Model 2	Model 3	Model 4	Model 5
**Emotional exhaustion**					
Adjusted R2	0.04	0.15	0.20	0.32	0.45
R2 change	0.04	0.11	0.05	0.12	0.13
F change statistics	38.14*	47.21*	45.46*	86.20*	75.68*
**Depersonalization**					
Adjusted R2	0.05	0.10	0.14	0.21	0.28
R2 change	0.05	0.05	0.04	0.07	0.07
F change statistics	47.38*	21.05*	34.76*	46.10*	27.54*

*p < 0.01.

*^(a)^Variables included: Model 1, demographic; Model 2, 1 + occupational; Model 3, 2 + socio-psychological; Model 4, 3 + intra-individual; and Model 5, 4 + organizational.*

The final model for emotional exhaustion showed that the predictive variables accounted for 45% of the variance. The analysis of the R^2^ changes revealed that the organizational and the intra-individual variables were the most relevant predictors, accounting for, respectively, 13 and 12% of the variance. On the contrary the results showed that the demographic, occupational, and socio-psychological sets of variables (accounting for, respectively, 11, 11, and 5% of the variance) encompassed no statistically significant predictors in the final step of the model.

A detailed analysis of the predictors in the final step of the model showed that the statistically significant determinants of emotional exhaustion were: the perceived organizational resources (*r* = −0.12), work schedule demands (*r* = 0.34), optimism (*r* = −0.12), and self-care (*r* = −0.15). Thus, considering the whole set of predictive variables included in the analysis, higher levels of emotional exhaustion were significantly associated with perceived lower levels of organizational resources, higher levels of work schedule demands, and lower levels of optimism and of self-care (and vice-versa).

The final model for depersonalization revealed that the predictive variables accounted for 28% of the variance. The analysis of the R^2^ changes showed that the demographic, intra-individual and organizational variables were the most relevant predictors, accounting for, respectively, 5, 7, and 7% of the variance, and all of them included statistically significant predictors in the final step of the model. The occupational and socio-psychological variables also explain 5 and 4%, respectively, of the variance but none of these sets of variables included statistically significant predictors in the final step of the model.

The detailed analysis of the predictors in the final step of the model showed that the statistically significant predictors of depersonalization were the perceived demands of the relationship with patients in general (*r* = 0.18), gender (*r* = −0.14), the perceived organizational resources (*r* = −0.13) and work schedule demands (*r* = 0.12), and problem-focused coping (*r* = −0.11). In other words, taking into account the all set of variables studied, higher levels of depersonalization were significantly associated with perceived higher levels of demands of the relationship with the patients in general and of the work schedule, lower levels of organizational resources and of problem-focused coping skills, and also with being a man (compared to a woman) (and vice-versa).

### Predictors of the Organizational Demands and Resources

Three stepwise hierarchic regressions were computed using the significant organizational predictors of the core burnout dimensions, namely the perceived organizational resources, demands of the relationship with the patients in general and work schedule demands, as dependent variables and all the previously considered demographic, occupational, socio-psychological, intra-individual, and organizational variables as predictors. As with the previous analysis, each one of the five sets of variables were added successively in the three regression models (Table 2; also see Supplementary Tables 5–7).

**TABLE 2:** Effect sizes for the hierarchical multiple regressions on organizational resources, demands of the relationship with the patients in general and work schedule demands.

	Model 1	Model 2	Model 3	Model 4	Model 5
**Organizational resources**					
Adjusted R2	0.03	0.22	0.41	0.46	0.53
R2 change	0.03	0.19	0.20	0.05	0.08
F change statistics	30.09*	87.36*	237.21*	39.02*	58.02*
**Demands of the relationship with the patients in general**					
Adjusted R2	0.08	0.21	0.21	0.29	0.41
R2 change	0.08	0.13	0.01	0.08	0.12
F change statistics	84.93*	56.35*	7.90*	53.38*	71.01*
**Work schedule demands**					
Adjusted R2	0.02	0.05	0.07	0.12	0.43
R2 change	0.02	0.04	0.02	0.06	0.30
F change statistics	15.75*	13.93*	13.33*	30.82*	188.68*

*p < 0.01.

The model for the perceived organizational resources, encompassing all the 29 predictive variables, accounted for 53% of the variance. The analysis of the R^2^ changes revealed that the occupational and socio-psychological variables were the most relevant, accounting for, respectively, 19 and 20% of the variance. Additionally, the demographic, intra-individual, and organizational variables accounted for, respectively, 3, 5, and 8% of the organizational resources explained variance. Importantly, only the occupational, socio-psychological and organizational sets of variables included statistically significant predictors in the final step of the model. The detailed analysis of the predictors in this final step of the model showed that the statistically significant determinants of the perceived organizational resources were procedural justice (*r* = 0.29), perceived demands due to the lack of autonomy (*r* = −0.20), working in a team (vs. individual) context (*r* = 0.15), perceived demands due to the lack of resources (*r* = −0.12). In sum, higher perceptions of organizational resources were significantly associated with higher perceived procedural justice, lower demands due to the lack of autonomy and to the lack of resources and working in a team (vs. individually) (and vice-versa).

The model for the perceived work schedule demands, comprising all the 29 predictive variables, accounted for 41% of the variance. The analysis of the R^2^ changes revealed that all sets of variables gave similar contributions for the amount of explained variance, ranging between 8 and 13%, with the exception of the socio-psychological variables that accounted solely for 1%. The detailed analysis of the predictors in the final step of the model showed that the statistically significant determinants of the perceptions about work schedule demands were the perception of mental demands (*r* = 0.24), self-care (*r* = −0.22), the perception of demands due to the lack of autonomy (*r* = 0.17) and the difference between effective and contracted working hours (*r* = 0.16). Hence, higher perceptions of work schedule demands were significantly associated with perceived higher mental demands and demands due to lack of autonomy, higher effective than contracted working hours and lower self-care (and vice-versa).

Finally, the model for the perception of demands of the relationship with patients in general, including all the 29 predictive variables, accounted for 43% of the variance. The analysis of the R^2^ changes revealed that the most relevant set of variables were the organizational’, accounting for 30% of the variance in the perception of demands of the relationship with patients in general. The results also showed that, along with the organizational variables, the demographic and occupational sets of variables included significant predictors in the final model. The detailed analysis of the predictors in this final step of the model showed that the statistically significant predictors were the perceived demands of the relationship with suffering patients (*r* = 0.42), the demands of the relationships in the workplace (*r* = 0.23), the number of years as specialist (*r* = −0.13), the age of the youngest child (*r* = −0.12), and the perceived demands due to the lack of resources (*r* = 0.11). Accordingly, higher perceptions of demands of the relationship with patients in general were significantly associated with perceived higher demands of the relationship with suffering patients, of the relationships in the workplace and due to the lack of resources, with the lower number of years as specialist and the lower age of the youngest child (and vice-versa).

## Discussion

Findings of large-scale studies (e.g., Shanafelt et al., 2012, 2015) point to physicians’ burnout as a public health problem (Schwenk and Gold, 2018) due to its purportedly alarming-level prevalence and negative costs for physicians’ occupational health, quality of patients’ care, and medical systems (e.g., Shanafelt et al., 2012, 2015; Panagioti et al., 2017). However, a recent systematic review (Rotenstein et al., 2018) revealed a large heterogeneity in prevalence estimates of physicians’ overall burnout and burnout’ symptoms thus hindering reliable conclusions. Also, a detailed characterization of burnout symptoms in Portuguese physicians is yet to be done, making it difficult to have an open dialogue and develop effective interventions in the specific context of Portugal. Accordingly, this study set out to characterize the burnout symptoms prevalence among Portuguese physicians.

Moreover and despite the importance of the burnout phenomena among physicians, a comprehensive study of the predictors of physicians’ burnout was still missing (Dyrbye et al., 2017; West et al., 2018), namely in Europe. This study provides evidence that contributes to fill this gap by studying a comprehensive, theory-driven set of factors influencing burnout in a large sample of Portuguese physicians. On a first set of analysis the hypothesis that perceived organizational demands and resources predict the core burnout symptoms (i.e., emotional exhaustion and depersonalization) on top of occupational, socio-psychological and intra-individual factors was tested. Additionally, a second set of analysis tested the predictive value of other context variables on the explanation of the organizational variables that contributed more to account for the burnout symptoms in the first set of analysis. This second set of analysis responds to the claim that more information besides physicians’ burnout prevalence and main predictors is needed to effectively address this phenomenon (Shanafelt et al., 2017). A more thorough understanding about the context antecedents of those organizational factors brings new insights on how to increase intervention potential (Nielsen and Maglia, 2017) in the prevention of burnout amongst physicians.

The results point to six important messages. First, there is a high-level of burnout symptoms among the Portuguese physicians. Second, organizational factors play a paramount role in accounting for burnout levels. In line with our prediction, the organizational factors, namely, the organizational resources, the demands of the work schedule, and the demands of the relationship with patients, predict burnout symptoms over and above the other factors considered herein. Additionally, the second set of analyses shows that these three organizational factors are also predicted by other organizational factors, with the same factors having second order effects. Third, optimism, self-care, and problem-focused coping are also important (negative) intra-individual predictors of burnout symptoms and open an important discussion about the role that individual physicians can and should have in monitoring and adapting their behaviors. Fourth, gender differences in depersonalization levels were found, consistently with the literature and reinforcing the importance of adjusting interventions and training with physicians accordingly. Fifth, although occupational and socio-psychological factors did not provide a significant account of the burnout symptoms, they do provide an important account of the organizational factors in the second set of analyses. Finally, issues regarding the measurement of burnout using MBI pointed us to the need to rethink its structure.

### The High Levels of Burnout Symptoms in Portuguese Physicians

Findings of the present study revealed that 66% of the participants reported a high level of Emotional exhaustion, approximately 39% a high level of Depersonalization and about 30% a high decrease in their Personal accomplishment, which may be considered alarming levels of burnout symptoms according to Shanafelt et al. (2012, 2015), and this scenario may worsen due to the present COVID-19 pandemic (Brooks et al., 2020).

Nevertheless, these results should not be used to define percentages of “burned-out” physicians for several reasons. While burnout has been included as an occupational phenomenon in the 10th Revision of the International Classification of Diseases (ICD-10) and, defined with more detail in the recent ICD-11, it is not classified as a medical condition [World Health Organization (WHO), 2019]. Statistical or clinical diagnostic criteria are needed to transform the continuous MBI scores into a dichotomous diagnostic. However, statistical cut-offs are based on frequency distributions and do not refer to clinical external criteria (Maslach et al., 1996; Schaufeli et al., 2009; Bianchi, 2017) and clinically validated cut-off points of the MBI are not available worldwide, namely for Portuguese physicians. Therefore, the MBI results of the present study should not be used to make dichotomous diagnoses of burnout and differentiate among “burned out” physicians and those who are not. This view is in line with the psychosocial perspective adopted in the present study that tends to define burnout as a socio-professional problem and calls for socio-professional responses to this problem (Schaufeli et al., 2009).

The lack of prior national data on Portuguese physicians’ burnout makes it unfeasible to put the results of the present study into historical context, and although they compare unfavorably with those of a study by Marcelino et al. (2012) focusing on Portuguese family doctors, as these two studies have targeted distinct populations it is not possible to ascertain whether the differences in results represent a worsening of the burnout rates over the last years.

The results of the present study also compare unfavorably with the findings of reference studies conducted nationwide in the United States with physicians of the various specialties (Shanafelt et al., 2012, 2015). Some changes occurring in the United States healthcare system and field of medicine, such as the reduction of employed physicians, the increasing clerical burden associated with the introduction of electronic health records, the financial pressures and the “unprecedented levels of scrutiny (quality metrics, patient satisfaction scores, measures of cost)” (Shanafelt et al., 2017, pp. 901), have been referred to as potential explanatory factors for the high burnout rates identified in United States physicians. A comparative cultural analysis between Portugal and the United States and their healthcare systems is beyond the scope of this work. Nevertheless, it is worth to mention that recent changes in patient needs, medical technology, and financial resources, coupled with austerity measures adopted in European countries such as Portugal, have also had significant negative effects on the availability of healthcare system resources, including reduced staffing and working conditions (Marques-Pinto et al., 2018). The COVID-19 pandemic impact on (Portuguese) physicians’ burnout is a challenge for future research.

### Burnout Symptoms as a Byproduct of Organizational Factors

This study set out to further the knowledge of factors that influence physicians’ burnout levels, and more specifically to test the hypothesis that perceived organizational demands and resources add a significant explanatory level of burnout symptoms on top of other factors (e.g., Linzer et al., 2001). The findings of the present study are in line with previous research within a psychosocial perspective (Schaufeli et al., 2009) of burnout (e.g., Linzer et al., 2001; Schaufeli and Buunk, 2003) and support our prediction. The results revealed that the organizational variables accounted for between one third and one quarter of the variance in emotional exhaustion and depersonalization. Importantly, both the organizational resources and the demands of the work schedule perceived by the physicians, and additionally the perceived demands of the relationship with patients, accounted for, respectively, emotional exhaustion, and depersonalization after considering the contribution of all the remaining predictors, namely of an intra-individual nature (i.e., personality factors such as optimism and locus of control, coping, and self-care behaviors).

The second set of analyses reinforces the importance of the organizational factors. In review, higher perceived demands due to lack of resources and due to lack of autonomy, perceived higher mental demands and demands due to lack of autonomy, and perceived higher demands of the relationship with suffering patients, of the relationships in the workplace and due to the lack of resources are among the most important predictors of, respectively, lower organizational resources, higher schedule demands, and higher demands of the relationship with patients in general. The primacy of organizational factors across the two sets of analyses highlights the importance of recognizing physicians’ burnout as a phenomenon that is, not only but also, the result of circumstancial factors that are, in principle, possible to address through interventions.

### An Individual Account of Burnout Symptoms

The results also showed that intra-individual variables also contributed to the explanation of emotional exhaustion, namely, optimism and self-care, and of depersonalization symptoms, namely, problem-focused coping, which remained statistically significant predictors in the final models of both regressions, giving support to and furthering the findings of previous studies. Optimism has been conceptualized as a personal resource in the Job Demands-Resources Model and some studies have confirmed its important role in maximizing engagement and reducing burnout symptoms, namely exhaustion (Xanthopoulou et al., 2007), in medical students (Hojat et al., 2015). Similarly, problem-focused coping has also been recognized as a fundamental component of individuals’ successful adaptation, with significant relations with reduced physicians’ emotional exhaustion and depersonalization (Lee et al., 2013). Also, more recent papers (e.g., West et al., 2016; Shanafelt et al., 2017; Jha et al., 2018) have acknowledged the important role that individual physicians may have in monitoring their own well-being, recognizing precocious signs of burnout, and adopting adequate self-care behaviors with a view to cope with burnout symptoms, and consequently to prevent a possible burnout – job stress vicious cycle (Guthier et al., 2020). Consistently with these conceptualizations and in line with other studies (e.g., Schaufeli and Bakker, 2004; Schaufeli and Taris, 2014), our results show that the lower the levels of organizational and personal resources and the higher the levels of demands perceived by the physicians, the higher their emotional exhaustion, and depersonalization. On the whole, these findings give support to previous theory assumptions, namely the more recent versions of the Job Demands–Resources model (e.g., Schaufeli and Taris, 2014), and to research outcomes on the relationships between organizational and personal resources (negative) and organizational demands (positive), and burnout (e.g., Schaufeli and Bakker, 2004; Schaufeli and Taris, 2014; Dyrbye et al., 2017).

The results of the present study also suggest interventions that aim at reducing mal-adaptive exhaustion and depersonalization through the stimulation of physicians’ optimism and self-care, and problem-focused coping, respectively. Indeed, as Bianchi et al. (2021) stated and their findings support, it may be “unwise” to de-emphasize individuals’ personality factors, such as neuroticism or the other Big Five traits, in burnout research, given the role they may have in the conceptualization, and effective prevention of burnout (Bianchi et al., 2021). Likewise, some authors have suggested that the personality profile of physicians may contribute to their propensity to burnout as it is expected that they are driven, competitive people, who can excel and must succeed at everything, while “Self-care is not a part of the physician’s professional training and typically is low on a physician’s list of priorities. Approximately one third of physicians do not have a doctor themselves.” Gundersen (2001, p. 146). Evidence from some reviews of literature and meta-analyses indicates that individual interventions aimed at enhancing self-awareness (e.g., mindfulness) and resilience skills have some success (West et al., 2016; Panagioti et al., 2017). Accordingly, Jha et al. (2018) advocate that access to health services for physicians, including mental health services, should urgently be improved. However, the focus on an individual approach to physicians’ burnout raises several concerns. From a practical point of view, it is unlikely that physicians would adhere or have the time to regularly and consistently include mindfulness and similar practices in their routine (Jha et al., 2018). And from a psychosocial principled perspective, a predominantly individual-level approach to intervention implies that burnout is the responsibility of individual physicians instead of a socio-professional problem (Shanafelt et al., 2017; Jha et al., 2018).

Alternatively, and in line with West et al. (2016) and Panagioti et al. (2017) reviews showing that the effects of organization-directed intervention programs on burnout were significantly larger than those of physician-directed interventions, the present study results suggest that the attempts to reduce burnout should address its root causes and operate at the individual-level mainly as a complement to broader organizational-level interventions. More specifically, improving the organizational resources available to physicians and diminishing the demands of the work schedule that physicians face may reduce significantly their emotional exhaustion and depersonalization, and diminishing the demands of the relationship with patients in general may also contribute to the reduction of this latter symptom of physicians’ burnout. However, as shown in the two aforementioned reviews of international literature, the majority of interventions tended to be physician-directed and the organization-directed interventions including measures such as rescheduling hourly shifts and reducing workload or strategies to enhance teamwork and leadership were remarkably rare (West et al., 2016; Panagioti et al., 2017). And this scenario is probably even more extreme in Portugal, after the austerity measures adopted in recent years and the consequent shortage of available healthcare system resources (Marques-Pinto et al., 2018), not to mention the COVID-19 pandemic impacts (Brooks et al., 2020).

### Gender Differences in Depersonalization

Gender was the sole demographic variable remaining a significant predictor in the final regression model on depersonalization. In line with general findings on physicians (e.g., Houkes et al., 2011), male physicians tended to score higher on depersonalization than their female colleagues. This tendency is more accentuated in female-typed occupations than in male-typed ones (Purvanova and Muros, 2010), which is the case of the medical profession in Portugal where men are underrepresented, as stated by the 2019 report of PORDATA (2019). According to the gender role theory, men, and women may experience burnout in different ways, as women are generally taught to display emotions and thus are more likely to express emotional exhaustion while men are generally encouraged to suppress emotions and consequently are more prone to psychological withdrawal, and depersonalization attitudes (Purvanova and Muros, 2010). As emotion-suppressive behaviors are usually seen as a sign of psychological adjustment, male physician’s burnout may go unrecognized and may not receive appropriate care (Wilcox, 1992). Hence, this result of the present study should be taken into account in interventions aimed at the professional development of medical students and practitioners.

### A Role for Procedural Justice and Teamwork in Reducing Burnout Symptoms

Occupational factors did not predict burnout symptoms in the final models of the regression. Recent reviews suggested that work-related stressors such as work hours, type of work, income or career stage fuel physicians’ burnout (e.g., Lee et al., 2013; Dyrbye et al., 2017; West et al., 2018). Other studies like the EGPRN (Soler et al., 2008) on European family doctors’ burnout, revealed some ambiguous results. Type of work (e.g., private) emerges as a significant predictor of emotional exhaustion and reduced personal accomplishment, and income was only weakly related with high burnout levels. In the present study none of the occupational variables studied were statistically significant predictors of emotional exhaustion and depersonalization when modeled together with the remaining groups of predictors. This may reflect the view that organizational factors have a greater influence on employee well-being than the occupational stressors that are peculiar to the professional group under investigation (Hart and Cooper, 2001). Although self-report questions were also used to gather the more factual data on occupational variables (e.g., private and/or public type of work) one cannot rule out that these findings may be contaminated by shared method variance problems, therefore additional studies using multiple data sources are essential for advancing the field (Donaldson and Grant-Vallone, 2002).

As for socio-psychological factors, the set of such variables included in the present study in the regression models, i.e., procedural justice, professional identity, and professional deprivation, again, did not make a meaningful contribution to the explained variance of emotional exhaustion or depersonalization, although similar factors have previously been found to predict physicians’ burnout symptoms (e.g., Smets et al., 2004; Jin et al., 2015; Jager et al., 2017). Indeed, while these factors have previously been found to predict burnout and wellbeing in organizational environments (Tyler, 1989; Trinkner et al., 2016; Andela and Truchot, 2017; Steffens et al., 2017), they were not examined within the framework of a larger model that also includes variables pertaining to demands (organizational) and resources (organizational and personal). The possibility that other socio-cultural factors may be important predictors of physicians’ burnout certainly should not be ruled out. As Schaufeli (2017) states, socio-cultural developments such as globalization, privatization, and liberalization that are pervasive in the United States and Europe may contribute to burnout as a social problem. In this vein, several authors from the United States analyze the new roots of this crisis in the “era of the electronic health record” in which the computer interfaces have disturbed physicians engagement with patients and autonomy with demands of documentation and quality measures, and call to action on physicians’ burnout (e.g., Jha et al., 2018). Hence, further research is needed to explore other socio-cultural factors of physicians’ burnout.

Although occupational and socio-psychological factors did not provide a significant account of the burnout symptoms, they do contribute to the understanding of the three organizational variables’ used in the second set of analysis. The results showed that higher procedural justice and teamwork (vs. individual), higher effective than contracted working hours, and having younger children and fewer years as specialist, are among the most important predictors of more favorable perceptions about, respectively, organizational resources, work schedule demands, and demands of the relationship with patients in general.

### Rethinking the Measurement of Burnout

A major issue with this work regards our use of a modified version of the MBI to measure each of the burnout dimensions. These transformations raise the concern about whether we are still talking about the same phenomena. Such transformations (e.g., the exclusion of items in each of the dimensions of burnout) have been occurring in empirical papers addressing burnout and with little discussion about what it means for the measurement of burnout. Taking in consideration that the studies with modified versions of the MBI keep amounting and that substantial modifications were needed even in a study like this one with close to 10,000 participants, this issue should no longer be ignored.

Burnout dimensions as measured by the MBI can be sensitive to contextual differences. In this sense, although from a psychometric point of view modifications are justifiable to assure that the different dimensions are indeed distinctive, it is important to develop and test theories on how and why contextual variables can influence the expression of burnout. Large psychometric studies of the MBI can be of great value here allowing to bridge theory with the specific feelings, attitudes and self-evaluations of MBI items.

## Conclusion

The findings of this study highlight the importance of recognizing physicians’ burnout as a complex phenomenon, with multiple-level factors that must be addressed through coordinated efforts at occupational, socio-psychological, individual, and organizational levels.

Some of the identified factors are fixed, unalterable, such as the age of the youngest child, the years as specialist, other are seen as insurmountable in times of austerity and crisis like the lack of resources, and other tend to be intrinsic to physicians professional practice for instance the mental demands and the demands of the relationship with suffering patients (e.g., Schaufeli et al., 2011). Nevertheless, some other factors may be subject to intervention efforts such as enhancing procedural justice, teamwork and level of participation in decision making, and reducing the effective working hours and stimulating physicians’ coping and self-care behaviors. Although these measures are seldom adopted (e.g., West et al., 2016; Panagioti et al., 2017), according to the results of the present study they may contribute to a more favorable perception of organizational resources and of work schedule demands, which in turn may influence lower levels of emotional exhaustion and depersonalization among these professionals. Additionally, improving the quality of the relationships in the workplace may also boost more positive perceptions about the relationships with patients in general and, consequently, further contribute to lower levels of depersonalization symptoms. In sum, “meaningful progress will require collaborative efforts by national bodies, health care organizations, leaders, and individual physicians, as each is responsible for factors that contribute to the problem and must own their part of the solution.” (Shanafelt et al., 2017, p. 2; for a thorough reflection on how to address physicians’ burnout see also e.g., Jha et al., 2018).

Despite its contributions, there are some limitations to the present study that should be taken into consideration. First, as this study relied on a cross-sectional design and was based solely on self-reports, inferences about correlational and causal relationships may be inflated by common method variance problems. The direction of the cross-sectional associations between job demands and emotional exhaustion, for instance, must be taken with caution, namely in light of the Guthier et al. (2020) meta-analysis results, showing that burnout more strongly predicts perceived job stressors than these predict burnout. Another example refers to the possible occurrence of measurement artifacts (Schaufeli and Enzmann, 1998) due to content overlap between the items of both types of measures (Bianchi et al., 2021). Thus, future research should consider using longitudinal designs and multiple sources of data set, also based on peer and supervisor ratings, and pay attention to measurement artifacts risks when selecting the assessment measures, with a view to minimize the validity threats of those method biases. Second, only 29% of physicians who received an invitation to participate in the study completed the MBI section of the survey. Although this response rate is higher than that of similar investigations and several studies did not find significant differences between responding physicians and those who do not respond (Shanafelt et al., 2012), it is not possible to assume that the study participants were representative of the Portuguese physicians population. Nevertheless, the comparisons made between the population, the study sample and an overlaid random sample showed very small differences regarding the distribution by gender, age, and regional affiliation to the OMP. Third, although the participants were physicians from the different specialties of medicine this study did not take this variable into consideration. Future studies should define how physicians’ burnout varies among the different specialties as they may experience and cope with its symptoms differently. Fourth, although some personality factors, such as optimism and locus of control, were tested as potential predictors of the core dimensions of burnout, the Big Five personality traits and specially neuroticism which has been strongly associated with burnout (e.g., Swider and Zimmerman, 2010; Bianchi et al., 2021), were not considered in the present study. This limitation should be addressed in future studies using multi-domain approaches and aiming at examining the nomological network of physicians’ burnout (Bianchi et al., 2021). Finally, it is important to reinforce that this study followed the long standing of research of burnout in western settings, so generalizability is limited. Cross-cultural research of this topic is highly in need.

Although this study does present some limitations, it explored burnout’ main predictors in a large sample drawn from all the regional sections of the OMP, in all specialties of medicine and practice settings and provides important clues for policy makers and human resources managers who have the opportunity to improve the work conditions of physicians in Portugal. Given the documented negative impacts of burnout on physicians’ well-being, the patients they serve and the health care system, further research is needed to design, implement, and evaluate the efficacy of coordinated occupational, socio-psychological, individual, and organizational interventions to prevent burnout in this professional group.

## Data Availability Statement

The datasets presented in this study can be found in online repositories. The names of the repository/repositories and accession number(s) can be found below: http://www.apis.ics.ulisboa.pt/apis0057/.

## Ethics Statement

The studies involving human participants were reviewed and approved by the Ordem dos Médicos Portuguesa. The patients/participants provided their written informed consent to participate in this study.

## Author Contributions

AM-P, SM, RC-L, NZ, and JV were involved in the initial conceptualization of the model and preparation of the survey. AM-P, SM, and RC-L were responsible for data analyses and first draft of the manuscript. JV was responsible for revision of the first draft. All authors contributed to the article and approved the submitted version.

## Conflict of Interest

The authors declare that the research was conducted in the absence of any commercial or financial relationships that could be construed as a potential conflict of interest.

## Publisher’s Note

All claims expressed in this article are solely those of the authors and do not necessarily represent those of their affiliated organizations, or those of the publisher, the editors and the reviewers. Any product that may be evaluated in this article, or claim that may be made by its manufacturer, is not guaranteed or endorsed by the publisher.
