# Supplementary material for: Predictors of Burnout Among Physicians: Evidence From a National Study in Portugal
Source: Front Psychol. 2021 Oct 1;12:699974. doi: 10.3389/fpsyg.2021.699974 (PMC8517183; doi:10.3389/fpsyg.2021.699974)
Supplement: Supplementary file 1 [file Table_1.DOCX]

Supplementary Material

# Supplementary Tables

Table 1. Comparisons of the distribution by gender, age and regional affiliation to OMP between the population, the study sample and an overlaid random sample.

| Variables | Population  N=43983 | Sample  n=9176 | Overlaid random sample  n=650 |
| --- | --- | --- | --- |
| Gender:  Female  Male | 53.4%  46.6% | 62.3%  37.7% | 60%  40% |
| Age group:  <30  31-35  36-40  41-45  46-50  51-55  56-60  61-65  >65 | 18.5%  10.9%  7.8%  6.6%  5.3%  9.0%  13.6%  13.0%  15.3% | 20.7%  16.7%  11.1%  8.1%  6.0%  10.2%  13.9%  10.1%  3.2% | 18.7%  16.9%  11.0%  7.2%  6.3%  9.9%  15.0%  10.6%  4.3% |
| Regional affiliation:  North  Center  South | 35.0%  16.9%  48.1% | 38.8%  19.6%  41.6% | 39.7%  17.9%  42.7% |

Table 2. Operationalization of the variables in the conceptual model and respective measures.

| Variables | Operationalization | Measurement |
| --- | --- | --- |
| Demographic | Gender | Male / Female |
|  | Relationship status | Living alone / Living with someone |
|  | Youngest child age | Year of birth of the |
| Occupational | Difference between effective and contracted working hours | Arithmetic difference between the self-reported estimate of the number of hours effectively worked in a week and the self-reported number of hours contracted |
|  | Compensatory rest after night shift | 0 – never to 10 – always |
|  | Years as specialist | Number of years |
|  | Professional stability | 0 – very unstable 10 – very stable |
|  | Individual vs. team work context | 0 – I manly work in an individual context to 10 – I manly work in a team context |
|  | Professional income | 0 – very difficult to live with my income to 10 – very easy to live with my income |
|  | Primary work place | Public / Private |
|  | Number of work places | Absolute number |
| Organizational | Organizational resources; demands of the relationship with suffering patients; mental demands; demands of the relationships in the workplace; work schedule demands; physical demands, demands due to the lack of resources; demands of the relationship with patients in general; and demands due to the lack of autonomy | Resources and Demands scale (reference) composed of 38 items measured on a Likert scale 0 – completely disagree to 10 – completely agree. EFA and CFA supported a 9 factor solution. |
| Intra-individual | Optimism | Items 4, 9 and 10 of the Life Orientation Test-Revised (LOT-R; Scheier & Carver, 1985; Scheier, Carver, & Bridges, 1994) measured from 0 – completely disagree to 10 – completely agree. EFA^1^ with a single factor: explained variance=50%, factorial weights between .37 and .87, and alpha=.69. |
|  | Locus of control | Items 13a, 13b, 28a and 28b of the reduced version of the Rotter's Locus of Control Scale (Rotter, 1966) measured from 0 – completely disagree to 10 – completely agree. EFA^1^ with a single factor: explained variance=35%, factorial weights between .50 and .71, alpha=.66. |
|  | Self-efficacy | Items 4, 5 and 10 of the Portuguese version of the Generalized Self-Efficacy Scale (Nunes, Schwarzer, & Jerusalem, 1999; Jerusalem & Schwarzer, 1992) measured from 0 – completely disagree to 10 – completely agree. EFA^1^ with a single factor: explained variance=72%, factorial weights between .78 and .93, alpha=.88. |
|  | Emotional regulation | Items 5 and 8 of the cognitive reappraisal and 4 and 9 of the emotional suppression subscales from the Portuguese version of the Emotion Regulation Questionnaire (ERQ; Vaz & Martins, 2008; Gross & John, 2003) measured from 0 – completely disagree to 10 – completely agree. Bivariate correlations indicate that only the cognitive reappraisal items have r >.30. |
|  | Problem-focused coping | Subscales of active coping and planification of the Portuguese version of Brief COPE (Pais Ribeiro & Rodrigues, 2004; Carver, 1997) measured from 0 – never do this to 10 – always do this. EFA^1^ with a single factor: explained variance=73%, factorial weights between .68 and .95, alpha=.91. |
|  | Self-care | Level of satisfaction with physical activity, leisure time, sleep quality and feeding (Chambers, Wakley, & Blenkinsopp, 2006; Renpenning & Taylor, 2011) measured from 0 – completely unsatisfied to 10 – completely satisfied. EFA^1^ with a single factor: explained variance=52%, factorial weights between .66 and .62, alpha=.81. |
| Social | Procedural justice | Single item adapted from the European Social Survey (ESS Round 7: European Social Survey, 2015) measured from 0 – completely disagree to 10 – completely agree. |
|  | Professional identity | Item 5 (centrality), 4 (ingroup affection) and 2 (ingroup bonding) of Cameron (2004) identity scale measured from 0 – completely disagree to 10 – completely agree. EFA^1^ with a single factor: explained variance=62%, factorial weights between .61 and .94, alpha=.80. |
|  | Professional deprivation:  respondent vs other physicians and specialty vs other specialties | Two items adapted from Relative Deprivation Scale (Lima & Vala, 2004) measured from 0 – clearly worst to 10 – clearly better. Bivariate correlation above the .30 criteria. |
| Burnout symptoms | Emotional exhaustion, Depersonalization and Personal accomplishment | Portuguese version of the Maslach Burnout Inventory – Human Service Survey (Marques Pinto, 2002; Maslach et al.,, 1996) with 23 items measured on a Likert scale 0 – never to 10 – every day  CFA^2^ with good adjustment for the tri-factorial version (factors for emotional exhaustion, depersonalization and professional realization; CFI of 0.95, TLI of 0.94, GFI of 0.96 and RMSEA of 0.07; items 9, 6, 10, 11, 12, 13, 14, 16, 19 and 21 removed based on the modification indexes), with alphas for each factor between .69 and .85 |

^1^ The statistical quality of the factors was granted using three criteria (Brown, 2006; Tabachnick & Fidel, 2009): factors’ eigenvalues >.7, factorial weights > .30, and communalities >.09. With the exception of the organizational resources and demands scale, the results of the EFAs all showed, as expected, unidimensional solutions with good levels of explained variance, factorial weights, and internal consistency . Due to the 2-item structure of the Emotional Regulation subscales, bivariate correlations were used to assess their psychometric performance and the same criteria as for the factorial weights, r<.30 was applied. As only the cognitive reappraisal items presented a correlation above the criteria, the emotional suppression items where dropped off the analysis.

^2^ The statistical quality of the CFA was assessed using two sets of criteria (Brown, 2006): first, measures of the overall goodness of fit, namely RMSEA < .07, Comparative Fit Index (CFI) and Tucker-Lewis Index (TLI) > .90; second, measures of localized areas of strain namely standardized residuals < 2.60 and general modification indexes < 5. The results for the first model revealed 10 problematic items. The results for the second solutions with the remaining 13 items reveled good adjustment to the three-factorial version (factors for emotional exhaustion, depersonalization and personal accomplishment). The scores for the factors identified in the CFA were computed using the regression method.

Table 3. Standardized estimates of the hierarchical multiple regression on emotional exhaustion.

|  | Model 1 | Model 2 | Model 3 | Model 4 | Model 5 |
| --- | --- | --- | --- | --- | --- |
| Gender | 0.07 | 0.08 | 0.08 | 0.05 | 0.03 |
| Relationship status | -0.05 | -0.06 | -0.03 | -0.02 | -0.01 |
| Youngest child | 0.18* | 0.22* | 0.14* | 0.08 | 0.01 |
| Difference between effective and contracted working time |  | 0.11* | 0.08 | 0.03 | -0.03 |
| Compensatory rest (after night shift in emergency) |  | -0.09 | -0.07 | -0.05 | -0.02 |
| Years as specialist |  | 0.10 | 0.01 | -0.01 | -0.01 |
| Professional stability |  | -0.13* | -0.08 | -0.01 | 0.00 |
| Individual versus group work context |  | -0.12* | -0.09 | -0.07 | -0.06 |
| Professional income |  | -0.16* | -0.12* | -0.07 | -0.03 |
| Primary work place |  | -0.07 | -0.07 | -0.04 | 0.02 |
| Number of work places |  | 0.01 | 0.01 | 0.02 | 0.00 |
| Procedural justice |  |  | -0.11* | -0.08 | -0.01 |
| Professional identity |  |  | -0.19* | -0.05 | -0.03 |
| Professional deprivation: respondent vs other physicians |  |  | 0.01 | -0.02 | -0.01 |
| Professional deprivation: respondent specialty vs other specialties |  |  | 0.03 | 0.02 | 0.00 |
| Optimism |  |  |  | -0.15* | -0.12* |
| Locus of control |  |  |  | -0.12* | -0.05 |
| Self-efficacy |  |  |  | -0.02 | -0.01 |
| Emotional Regulation |  |  |  | 0.01 | 0.00 |
| Problem solving |  |  |  | 0.03 | 0.01 |
| Self-care |  |  |  | -0.27* | -0.15* |
| Organizational Resources |  |  |  |  | -0.12* |
| Demands of the relation with patients in suffering |  |  |  |  | 0.04 |
| Mental demands |  |  |  |  | -0.01 |
| Demands of the relations in the workplace |  |  |  |  | 0.01 |
| Schedule demands |  |  |  |  | 0.34* |
| Physical demands |  |  |  |  | 0.04 |
| Demands due to the lack of resources |  |  |  |  | 0.00 |
| Demands of the relation with patients in general |  |  |  |  | 0.08 |
| Demands due to the lack of autonomy |  |  |  |  | 0.05 |
| Adjusted R2 | 0.04 | 0.15 | 0.20 | 0.32 | 0.45 |
| R2 change | 0.04 | 0.11 | 0.05 | 0.12 | 0.13 |
| F change statistics | 38.14* | 47.21* | 45.46* | 86.20* | 75.68* |

Note: Only β > .10 were tested for significance; *p < .01.

Table 4. Standardized estimates of the hierarchical multiple regression on depersonalization.

|  | Model 1 | Model 2 | Model 3 | Model 4 | Model 5 |
| --- | --- | --- | --- | --- | --- |
| Gender | -0.12* | -0.12* | -0.12* | -0.13* | -0.14* |
| Relationship status | -0.09 | -0.06 | -0.03 | -0.03 | -0.02 |
| Youngest child | 0.21* | 0.13* | 0.06 | 0.06 | 0.05 |
| Difference between effective and contracted working time |  | 0.04 | 0.02 | 0.01 | 0.00 |
| Compensatory rest (after night shift in emergency) |  | -0.04 | -0.02 | -0.02 | 0.00 |
| Years as specialist |  | -0.05 | -0.14* | -0.11* | -0.08 |
| Professional stability |  | -0.09 | -0.05 | 0.01 | 0.03 |
| Individual versus group work context |  | -0.11* | -0.09 | -0.07 | -0.05 |
| Professional income |  | -0.08 | -0.05 | -0.02 | 0.01 |
| Primary work place |  | -0.10* | -0.09 | -0.07 | -0.02 |
| Number of work places |  | 0.01 | 0.01 | 0.03 | 0.02 |
| Procedural justice |  |  | -0.09 | -0.06 | 0.00 |
| Professional identity |  |  | -0.19* | -0.06 | -0.03 |
| Professional deprivation: respondent vs other physicians |  |  | -0.02 | -0.03 | -0.03 |
| Professional deprivation: respondent specialty vs other specialties |  |  | 0.01 | 0.01 | -0.01 |
| Optimism |  |  |  | -0.09 | -0.07 |
| Locus of control |  |  |  | -0.11* | -0.06 |
| Self-efficacy |  |  |  | -0.05 | -0.03 |
| Emotional Regulation |  |  |  | -0.01 | -0.02 |
| Problem solving |  |  |  | -0.13* | -0.11* |
| Self-care |  |  |  | -0.08 | -0.03 |
| Organizational Resources |  |  |  |  | -0.13* |
| Demands of the relation with patients in suffering |  |  |  |  | -0.07 |
| Mental demands |  |  |  |  | -0.04 |
| Demands of the relations in the workplace |  |  |  |  | -0.03 |
| Schedule demands |  |  |  |  | 0.12* |
| Physical demands |  |  |  |  | 0.03 |
| Demands due to the lack of resources |  |  |  |  | 0.02 |
| Demands of the relation with patients in general |  |  |  |  | 0.18* |
| Demands due to the lack of autonomy |  |  |  |  | 0.07 |
| Adjusted R2 | 0.05 | 0.10 | 0.14 | 0.21 | 0.28 |
| R2 change | 0.05 | 0.05 | 0.04 | 0.08 | 0.06 |
| F change statistics | 47.38* | 21.05* | 34.76* | 46.10* | 27.54* |

Note: Only β > .10 were tested for significance; *p < .01.

Table 5. Standardized estimates of the hierarchical multiple regression on organizational resources.

|  | Model 1 | Model 2 | Model 3 | Model 4 | Model 5 |
| --- | --- | --- | --- | --- | --- |
| Gender | -0.01 | -0.01 | 0.00 | 0.01 | 0.00 |
| Relationship status | 0.07 | 0.07 | 0.01 | 0.01 | 0.00 |
| Youngest child | -0.17* | -0.17* | -0.03 | -0.04 | -0.03 |
| Difference between effective and contracted working time |  | -0.04 | 0.02 | 0.03 | 0.04 |
| Compensatory rest (after night shift in emergency) |  | 0.07 | 0.04 | 0.03 | 0.01 |
| Years as specialist |  | -0.06 | 0.07 | 0.05 | 0.02 |
| Professional stability |  | 0.24* | 0.15* | 0.10 | 0.08 |
| Individual versus group work context |  | 0.19* | 0.15* | 0.13* | 0.15* |
| Professional income |  | 0.15* | 0.09 | 0.07 | 0.04 |
| Primary work place |  | 0.14* | 0.13* | 0.11* | 0.05 |
| Number of work places |  | 0.00 | 0.01 | -0.01 | -0.02 |
| Procedural justice |  |  | 0.38* | 0.36* | 0.29* |
| Professional identity |  |  | 0.22* | 0.13* | 0.09 |
| Professional deprivation: respondent vs other physicians |  |  | -0.02 | 0.00 | -0.01 |
| Professional deprivation: respondent specialty vs other specialties |  |  | -0.04 | -0.03 | -0.02 |
| Optimism |  |  |  | 0.08 | 0.07 |
| Locus of control |  |  |  | 0.07 | 0.03 |
| Self-efficacy |  |  |  | 0.09 | 0.10 |
| Emotional Regulation |  |  |  | 0.02 | 0.03 |
| Problem solving |  |  |  | 0.05 | 0.01 |
| Self-care |  |  |  | 0.05 | 0.04 |
| Demands of the relation with patients in suffering |  |  |  |  | 0.09 |
| Mental demands |  |  |  |  | 0.06 |
| Demands of the relations in the workplace |  |  |  |  | -0.05 |
| Schedule demands |  |  |  |  | 0.00 |
| Physical demands |  |  |  |  | -0.09 |
| Demands due to the lack of resources |  |  |  |  | -0.12* |
| Demands of the relation with patients in general |  |  |  |  | 0.01 |
| Demands due to the lack of autonomy |  |  |  |  | -0.20* |
| Adjusted R2 | 0.03 | 0.22 | 0.41 | 0.46 | 0.53 |
| R2 change | 0.03 | 0.19 | 0.20 | 0.05 | 0.08 |
| F change statistics | 30.09* | 87.36* | 237.21* | 39.02* | 58.02* |

Note: Only β > .10 are tested for significance; *p < .01.

Table 6. Standardized estimates of the hierarchical multiple regression on schedule demands.

|  | Model 1 | Model 2 | Model 3 | Model 4 | Model 5 |
| --- | --- | --- | --- | --- | --- |
| Gender | 0.05 | 0.07 | 0.07 | 0.05 | 0.03 |
| Relationship status | -0.04 | -0.05 | -0.04 | -0.03 | -0.04 |
| Youngest child | 0.28* | 0.33* | 0.30* | 0.22* | 0.20* |
| Difference between effective and contracted working time |  | 0.26* | 0.25* | 0.19* | 0.16* |
| Compensatory rest (after night shift in emergency) |  | -0.10* | -0.10 | -0.08 | -0.06 |
| Years as specialist |  | 0.12* | 0.09 | 0.06 | 0.07 |
| Professional stability |  | -0.02 | -0.01 | 0.03 | 0.05 |
| Individual versus group work context |  | 0.01 | 0.02 | 0.02 | 0.00 |
| Professional income |  | -0.12* | -0.11* | -0.07 | -0.04 |
| Primary work place |  | -0.13* | -0.13* | -0.12* | -0.07 |
| Number of work places |  | 0.08 | 0.08 | 0.08 | 0.08 |
| Procedural justice |  |  | -0.04 | -0.02 | 0.04 |
| Professional identity |  |  | -0.07 | 0.02 | 0.03 |
| Professional deprivation: respondent vs other physicians |  |  | -0.02 | -0.04 | -0.02 |
| Professional deprivation: respondent specialty vs other specialties |  |  | 0.05 | 0.04 | 0.03 |
| Optimism |  |  |  | -0.03 | -0.01 |
| Locus of control |  |  |  | -0.11* | -0.05 |
| Self-efficacy |  |  |  | 0.03 | 0.02 |
| Emotional Regulation |  |  |  | 0.02 | 0.00 |
| Problem solving |  |  |  | 0.07 | 0.03 |
| Self-care |  |  |  | -0.28* | -0.22* |
| Organizational Resources |  |  |  |  | 0.00 |
| Demands of the relation with patients in suffering |  |  |  |  | 0.02 |
| Mental demands |  |  |  |  | 0.24* |
| Demands of the relations in the workplace |  |  |  |  | 0.05 |
| Physical demands |  |  |  |  | 0.03 |
| Demands due to the lack of resources |  |  |  |  | 0.06 |
| Demands of the relation with patients in general |  |  |  |  | 0.05 |
| Demands due to the lack of autonomy |  |  |  |  | 0.17* |
| Adjusted R2 | 0.08 | 0.21 | 0.21 | 0.29 | 0.41 |
| R2 change | 0.08 | 0.13 | 0.01 | 0.08 | 0.12 |
| F change statistics | 84.93* | 56.35* | 7.90* | 53.38* | 71.01* |

Note: Only β > .10 were tested for significance; *p < .01.

Table 7. Standardized estimates of the hierarchical multiple regression on demands of the relationship with patients in general.

|  | Model 1 | Model 2 | Model 3 | Model 4 | Model 5 |
| --- | --- | --- | --- | --- | --- |
| Gender | 0.12* | 0.11* | 0.11* | 0.09 | 0.01 |
| Relationship status | -0.03 | 0.02 | 0.04 | 0.04 | 0.02 |
| Youngest child | 0.03 | -0.08 | -0.12* | -0.12* | -0.12* |
| Difference between effective and contracted working time |  | -0.01 | -0.02 | -0.05 | -0.04 |
| Compensatory rest (after night shift in emergency) |  | -0.01 | -0.01 | 0.00 | 0.01 |
| Years as specialist |  | -0.10* | -0.16* | -0.14* | -0.13* |
| Professional stability |  | -0.11* | -0.08 | -0.03 | -0.02 |
| Individual versus group work context |  | -0.07 | -0.06 | -0.05 | -0.04 |
| Professional income |  | -0.07 | -0.05 | -0.02 | 0.00 |
| Primary work place |  | -0.05 | -0.05 | -0.03 | 0.01 |
| Number of work places |  | -0.03 | -0.03 | -0.01 | 0.00 |
| Procedural justice |  |  | -0.03 | -0.01 | 0.07 |
| Professional identity |  |  | -0.12* | -0.03 | -0.03 |
| Professional deprivation: respondent vs other physicians |  |  | 0.01 | -0.01 | -0.01 |
| Professional deprivation: respondent specialty vs other specialties |  |  | 0.05 | 0.05 | 0.04 |
| Optimism |  |  |  | -0.07 | -0.02 |
| Locus of control |  |  |  | -0.14* | -0.05 |
| Self-efficacy |  |  |  | -0.09 | -0.04 |
| Emotional Regulation |  |  |  | 0.05 | 0.01 |
| Problem solving |  |  |  | 0.03 | -0.02 |
| Self-care |  |  |  | -0.10 | -0.01 |
| Organizational Resources |  |  |  |  | 0.01 |
| Demands of the relation with patients in suffering |  |  |  |  | 0.42* |
| Mental demands |  |  |  |  | 0.03 |
| Demands of the relations in the workplace |  |  |  |  | 0.23* |
| Schedule demands |  |  |  |  | 0.04 |
| Physical demands |  |  |  |  | 0.02 |
| Demands due to the lack of resources |  |  |  |  | 0.11* |
| Demands due to the lack of autonomy |  |  |  |  | 0.08 |
| Adjusted R2 | 0.02 | 0.05 | 0.07 | 0.12 | 0.43 |
| R2 change | 0.02 | 0.04 | 0.02 | 0.06 | 0.30 |
| F change statistics | 15.75* | 13.93* | 13.33* | 30.82* | 188.68* |

Note: Only β > .10 are tested for significance; *p < .01.
